# PPARs in Viral Disease

**DOI:** 10.1155/2009/393408

**Published:** 2009-07-14

**Authors:** Jacqueline Capeau, Lawrence Serfaty, Mostafa Badr

**Affiliations:** ^1^INSERM, U938, CDR Saint-Antoine, 75012 Paris, France; ^2^UPMC University Paris 06, UMR_S 938, CDR Saint-Antoine, 75012 Paris, France; ^3^AP-HP Tenon and Saint-Antoine Hospitals, 75012 Paris, France; ^4^Division of Pharmacology and Toxicology, University of Missouri-Kansas City, Kansas City, MO 64108, USA

This special issue of PPAR Research contains an exciting array of valuable reviews examining the relationship between PPARs and various viral infections, varying from HIV to HCV and HBV. This assembly of reviews includes a group of three complimentary reviews addressing different facets of the cross-talk between HIV infection and antiretroviral treatment as well as the potential beneficial role of PPAR*γ* in HIV-associated lipodystrophy. First, Giralt et al. detail the cross-talk between HIV infection and PPAR*γ* leading to the reported negative impact of this interplay on adipose tissue physiology. They also present literature on the reciprocal, yet contradictory roles of PPAR*α* and PPAR*γ* on HIV-1 replication and transcription. Second, Caron et al. describe the effect of ART on adipocyte PPAR*γ* expression in vitro, in animal models, and in HIV-infected patients. These authors also report on the intriguing connection between PPAR*γ*, activated macrophages found in the adipose tissue and ART. In the third review of this series, J. Sutinen closely examines the results of 14 different clinical trials which evaluated if the PPAR*γ* thiazolidinedione agonists could be useful in the treatment of HAART-associated metabolic complications in HIV-infected patients. Upon careful evaluation of the reported results, Sutinen concludes that these agonists produce a very modest, if any, effect on lipoatrophic subcutaneous adipose tissue, despite their improvement of insulin sensitivity in treated patients. The review by Doran and coworkers focuses on the studies dealing with the potential role of PPAR*γ* in HIV-1-associated bone disease. These authors put forth a provocative hypothesis which stipulates a potential role for PPAR*γ* in the reduced bone mass associated with HIV-1 infection and treatment. Specifically, they suggest a possible dysregulation of the activity of PPAR*γ* in undifferentiated stromal cells or in partially differentiated preosteoblast and preadipocyte cells. The liver which is impacted not only by HIV but also by HBV and HCV is the focus of several excellent reviews in this special issue. In a review by Lemoine and coauthors, the role that PPARs play in HIV infection, in terms of associated metabolic disorders, disease progression, coinfections with HBV or HCV, and response to antiviral treatment, is featured. In addition, the summary by Lemoine et al. of the experimental and clinical data regarding PPARS in HIV-associated liver disease provides a rationale for the use of PPAR*γ* agonists as therapeutic agents in these patients. Further, Negro reviews experimental and clinical data suggesting that HCV may interfere with hepatic insulin signaling, possibly involving the downregulation of the PPAR*γ*. Capitalizing on the known involvement of PPARs in lipid metabolism, inflammatory process, and fibrogenesis, Dharancy et al. have summarized experimental and human studies showing a diminished expression and function of PPAR*α* and PPAR*γ* during HCV infection. These authors also review the potential benefits of nonhepatotoxic PPAR*α*/*γ* agonists as therapeutic agents to treat chronic hepatitis C. In another valuable review by Dubuquoy and coauthors, the potential of PPARs to modulate HBV transcription and replication is summarized. The authors report that, in HBV transgenic mouse model, activation of PPAR*α* increased the transcription and replication of HBV, thus concluding that modulating the PPAR*α*/RXR heterodimer may be an interesting therapeutic option to control HBV infection. We are assured that the reviews presented in this special issue, on the interplay between PPARs and viral disease, will be highly useful for those with interest in the field.

*Jacqueline Capeau* *Jacqueline Capeau* *Lawrence Serfaty* *Lawrence Serfaty* *Mostafa Badr* *Mostafa Badr*

